# PtCP1 Is an Extraplastidial Cysteine Protease Involved in Leaf Protein Degradation of *Populus tomentosa Carr*

**DOI:** 10.3390/plants15101530

**Published:** 2026-05-16

**Authors:** Yawei Fan, Jingyi Han, Xiatong Liu, Han Liu, Mengyu Zhang, Xincaiyu Cui, Hui Li, Hai Lu

**Affiliations:** 1 State Key Laboratory of Tree Genetics and Breeding, The Tree and Ornamental Plant Breeding and Biotechnology Laboratory of National Forestry and Grassland Administration, College of Biological Sciences and Biotechnology, Beijing Forestry University, Beijing 100083, China; fanyawei13@163.com (Y.F.); jingyihan@bjfu.edu.cn (J.H.); liuhan291999@163.com (H.L.); zhangmengyu202103@163.com (M.Z.); cuixincaiyu@163.com (X.C.); 830lihui@163.com (H.L.); 2 Key Laboratory of State Forestry and Grassland Administration on Desert Oasis Ecosystem Protection and Restoration, Xingjiang Key Laboratory of Fruit Tree Species Breeding and Cultivation, Xinjiang Key Laboratory of Forestry and Grassland Sand Control and Desert Industry, Xinjiang Academy of Forestry, Urumqi 830063, China

**Keywords:** CRISPR/Cas9, cysteine protease, populus tomentosa, rubisco turnover, biomass accumulation

## Abstract

Protein turnover is essential for cellular metabolism, organelle biogenesis, stress adaptation, and ultimately the viability of cells and tissues. Papain-like cysteine proteases (PLCPs) are one of the vital components in protein degradation. PLCPs have been reported to act in senescence-associated proteolysis, but their roles in vegetative growth remain unclear. We identified PtCP1, an AALP-like PLCP in *Populus tomentosa*, localized to the vacuole and acid-triggered activated. CRISPR/Cas9-generated loss-of-function mutant (*d7*) showed dwarfism and non-stomatal photosynthetic limitations. On the other hand, the gain-of-function line (EM, deleted ERFNIN domain) exhibited accelerated growth and enhanced photosynthetic parameters. We showed *d7* had the accumulation of Rubisco, which was the most important protein in photosynthetic carbon fixation. Transcriptomics revealed dysregulated carbon metabolism in *d7*. This data supported *PtCP1*-mediated proteolysis regulated photosynthetic carbon assimilation via altered Rubisco turnover, and then it increased the biomass accumulation during vegetative growth in woody plants.

## 1. Introduction

Proteostasis—the maintenance of a functional and healthy proteome—is essential for cellular metabolism, organelle biogenesis, stress adaptation, and ultimately the viability of cells and tissues. As cells constantly face internal fluctuations and external stresses, the proteome must be continuously remodeled, with damaged or misfolded proteins being selectively repaired or eliminated. Protein degradation in eukaryotic cells is predominantly governed by the ubiquitin–proteasome system (UPS) alongside the autophagy–lysosome pathway (ALP) [1,2]. In plants, proteostasis is maintained primarily by the UPS and a vacuolar degradation pathway, where cysteine proteases act as vital players.

The expression and activity of cysteine protein are strictly regulated by a complex network in response to diverse intrinsic and exogenous factors, including aging, plant hormones, lighting, drought, frost, pathogens, and so on. Papain-like cysteine proteases (PLCPs) are classified within the CA superfamily (family C1) of the MEROPS database and are characterized by a catalytic triad (Cys-His-Asn) structurally similar to papain [3]. To prevent unwanted proteolysis, they are synthesized as inactive precursors, with maturation precisely controlled both temporally and spatially [4,5]. This regulation involves inhibitory prodomains and motifs like ERFNIN, which mediates an acidic pH-dependent activation mechanism ensuring their activity is deployed at the appropriate time and location [6,7,8].

While cysteine proteases are well-established mediators of protein degradation during senescence, emerging evidence indicates their involvement in chloroplast protein turnover during vegetative growth as well. Cysteine proteases have been implicated in chloroplast protein turnover during plant growth. A previous study showed that the overexpression of the rice cysteine protease inhibitor Oryzacystatin-I (OC-I) in Arabidopsis thaliana led to more biomass accumulation and higher soluble protein content in transgenic lines after flowering [9]. Similarly, OC-I overexpression in tobacco delayed senescence-associated declines in photosynthesis activity and increased plant biomass and leaf protein content after flowering [10]. These results also indicated the increased biomass mediated by RuBisCO (ribulose-1,5-bisphosphate carboxylase/oxygenase) accumulation.

RuBisCO (ribulose-1,5-bisphosphate carboxylase/oxygenase), the most abundant protein on Earth, catalyzes the initial and rate-limiting step of photosynthetic carbon fixation in the Calvin–Benson–Bassham cycle. It facilitates the carboxylation of ribulose-1,5-bisphosphate (RuBP), converting atmospheric CO_2_ into two molecules of 3-phosphoglycerate (3-PGA). This reaction represents the core of biological carbon cycling, accounting for the assimilation of approximately 100 gigatons of carbon annually into biomass globally [11,12]. In plants, Form I RuBisCO consists of eight large (rbcL) and eight small (RbcS) subunits, and its function is modulated by assembly factors and metabolic feedback; for example, sugar accumulation represses its expression and activity [13,14].

Accumulating evidence indicates that PLCPs participate in the degradation of chloroplast proteins, particularly rubisco. The barley cysteine protease HvPAP14 has been demonstrated to cleave LHCB proteins, PSBO, and the large subunit of Rubisco within chloroplasts [15]. This partial degradation of Rubisco by HvPAP14 likely occurs inside chloroplasts prior to the formation of Rubisco-containing vesicles [10] and autophagic processes [16]. SAG12 (senescence-associated gene 12), the most extensively studied cysteine protease, is localized in acidic senescence-associated vacuoles and has been directly implicated in Rubisco degradation [17,18]. These findings collectively establish the crucial role of PLCPs in chloroplast protein degradation.

Based on these studies, we hypothesized that additional cysteine proteases, beyond those already implicated in senescence, may actively regulate protein turnover and carbon metabolism during vegetative growth in woody plants. To test this hypothesis, we identified an AALP-like cysteine protease, *PtCP1*, in *Populus tomentosa*. The aim of this study was to determine the function of PtCP1 in plant growth.

## 2. Materials and Methods

### 2.1. Plant Materials and Growth Conditions

Plants of the poplar hybrid 741 ([*Populus alba* × (*P. davidiana* + *P. simonii*) × *P. tomentosa*]) (month-old) were grown on solid 1/2 MS medium, pH 6.15, in a growth chamber with a 14 h light/10 h dark photoperiod at 25 °C. They were transplanted in seed pots (10 × 10 cm^2^) in the phytotron artificial climate chamber with a 16 h day/8 h night cycle (22–25 °C, 60% humidity, 54 μmol m^−2^ s^−1^) in Beijing Forestry University, Beijing, China. The plants were watered every 3 days for 60 days before treatment.

### 2.2. Molecular Cloning and Plasmid Construction

Full-length cDNA was amplified with the primer pair *PtCP1-F/R* and cloned into pMD-18T for the sequencing assays. A 3.06 kb genomic DNA fragment containing the *PtCP1* genomic sequence and the Pro*PtCP1* promoter was amplified with the Pro*PtCP1-F/R* primers and inserted into the pBI121 binary vector for the pro*PtCP1*::GUS. The open reading frame (ORF) minus the first 66 bp of *PtCP1* cDNA was amplified by PCR with the two *PtCP1-E-F/R* primers and inserted downstream of the pET30a plasmid (Qiagen, Hilden, Germany) T7 promoter. The expression, extraction, purification, and renaturation of the PtCP1 protein were performed according to the procedure described by Zhang et al. [19].

### 2.3. Bio-Informatic Prediction and Analyses

The methods and the PLCP protein sequences used for multiple alignments and phylogenetic tree analysis were as described by Liu et al. [20]. Protein signal peptides were predicted using the SignalP4.1 server (http://www.cbs.dtu.dk/services/SignalP/ (accessed on 9 June 2022)). The protein structure was predicted using Swiss-model (https://swissmodel.expasy.org (accessed on 9 June 2022)).

### 2.4. RNA Extraction and qRT-qPCR Analysis

Total RNA was extracted and cDNA was synthesized following the instructions on a plant total RNA extraction kit (Tiangen, Beijing, China) and a Fasting RT Kit (Tiangen). PtCP1 expression in different tissues was assessed by qRT-PCR using the qRT-*PtCP1*-F/R primers. Actin was used as a control with the Actin-F/R primer. The qRT-qPCR analyses were performed using SYBR Green qPCR Mix (Tiangen) on an CFX Connect Real-Time PCR detection system (Bio-Rad Laboratories, Hercules, CA, USA). The PCR conditions were 94 °C for 3 min, 40 cycles at 94 °C for 10 s, 55 °C for 20 s, 72 °C for 20 s, 60 °C for 30 s, and 72 °C for 1 min. Data were analyzed using the Bio-Rad CFX Maestro 1.1 (Version: 4.1.2433.1219, Bio-Rad Laboratories) software, and the differences in gene expression were calculated using the 2^−ΔΔCt^ method. Primer information was provided in Supplemental Table S1.

### 2.5. GUS Assay

The pBI121 binary vector used to express pro*PtCP1*::GUS was transformed into *Agrobacterium tumefaciens* strain GV3101 before infecting tobacco leaves. The *Agrobacterium*-mediated transformation was performed. The materials were selected using kanamycin. PCR was performed using GUS-F/R primers to validate the transgenic plants at the DNA level. The transgenic lines were treated with 90% (*v*/*v*) pre-cooled acetone for 1 h, stained with X-Gluc solution, and incubated at 37 °C for 12 h to visualize GUS activity.

### 2.6. Purification of Protease and Assay of Protease Activity

The recombinant pET30a plasmid carrying the zymogen of PtCP1 was transformed into *E. coli* BL21 cells, and positive colonies were confirmed by sequencing. The zymogens of PtCP1 were produced in *E. coli* as inclusion bodies. The inclusion bodies were washed and resuspended in solution S (50 mM Tris-HCl, 1 mM EDTA, 0.5% Triton X-100, and 0.15% 2-hydroxy-1-ethanethiol, pH 8.0) containing 2 M, 4 M, 6 M, and 8 M urea. Protein renaturation was performed by dialyzing in solution R [50 mM Tris-HCl, 1 mM EDTA, 1 mM glutathione, 0.1 mM glutathione (oxidized)] containing 8 M, 6 M, 4 M, and 2 M urea at pH 8.0 and 4 °C. For self-activation, the zymogen of the PtCP1 enzyme (0.1 mg) was mixed with 20 μL of buffer (0.1 M NaH_2_PO_4_, 0.1 M Na_2_HPO_4_, 0.1 M NaCl, 30 mM L-cysteine, and 6 mM EDTA, pH 3.0) and incubated for 30 min at 37 °C. The methods for the assay of protease activity and the optimum temperature, the optimum pH, and the effect of inhibitors on protease activity were used, referring to the research conducted by Yang et al. [21]. The sample and protein marker were loaded on a 12% SDS-PAGE gel to verify the protein.

### 2.7. Paraffin Sections

The last stem nodes from 3-month-old WT (wild type) and transgenic plants were fixed in glutaraldehyde fixation solution (2.5% glutaraldehyde and 0.1 M phosphate-buffered saline (PBS), pH 7.4) for 12 h before being dehydrated in an alcohol gradient series (30 min each in 50%, 70%, 95%, and 100% alcohol) to prepare paraffin sections. The sections were cleared in xylene/alcohol gradient series. The samples were sequentially embedded in paraffin at 62 °C for 2 days. The paraffin-embedded sections measuring 800 nm were cut using a UC6 ultramicrotome (Leica, Wetzlar, Germany), stained with 1% toluidine blue O (Sigma–Aldrich, St. Louis, MO, USA), and photographed using a Leica DM 2500 LED microscope.

### 2.8. PtCP1 Immunolocalization

A protein-specific polypeptide (aa 2–13) (224–235 aa of PtCP1): C*TEEAYPYTGKDD (C*: artificially added cysteine for the conjugation of KLH) as an antigen epitope was selected as an immunogen to develop an anti-PtCP1 polyclonal antibody by immunizing the New Zealand white rabbit (BGI, Beijing, China). The antibody specificity was examined by immunoblotting using PtCP1 *E. coli* recombinant proteins. The specificity of the anti-PtCP1 antibody was confirmed by hybridization with a membrane blotted with protein extracts from the *E. coli* recombinant proteins. WT *Populus* leaves were fixed and embedded as described by Schmid et al. [22]. The sections were then labeled, examined, and photographed as described by Zhang et al. [23].

### 2.9. Biochemical Assays

The leaf samples collected from the 3-month-old WT and transgenic tissue of cultured *P. tomentosa* were prepared for isolating soluble proteins. The samples with the same dry weight were homogenized in PBS, and soluble proteins were isolated by centrifugation (4 °C, 10,000 rpm, 10 min) (Beckman Allegra X-30R, rotor F0850, Beckman Coulter, Inc., Brea, CA, USA). The soluble protein concentration in the supernatant was determined using a bicinchoninic acid (BCA) protein assay kit (Pierce BCA Protein Assay Kit, Thermo Scientific, Waltham, MA, USA) following the manufacturer’s protocols. Soluble proteins (5 μg) were subjected to SDS-PAGE on 12.5% (*w*/*v*) gels containing 0.1% (*w*/*v*) SDS; the gels were stained with Coomassie brilliant blue. Total protein extracted from the leaves of *P. tomentosa* was immunolabeled with primary antibodies anti-PtCP1, anti-β-actin (Catalog: bsm-33128M, Bioss, Beijing, China), and anti-RuBisCo (Catalog: OHY0066A, PhytoAB, Beijing, China) antibody at a dilution of 1:2000 and a secondary goat anti-rabbit IgG (Catalog: BN20604, Biorigen, Beijing, China) and goat anti-mouse IgG antibody (Catalog: BN20601, Biorigen, Beijing, China) conjugated to horseradish peroxidase at a dilution of 1:5000. Luminescence was detected using the Immobilon Western Chemiluminescent HRP Substrates (Millipore, Burlington, MA, USA). The results were photographed using a ChemiDocTM MP Imaging System (Bio-Rad, CA, USA).

### 2.10. Generation of Transgenic Plants

A CRISPR/Cas9 multiplex target editing site system was used for multiplex editing as described by Xie et al. [24]. Based on the DNA sequence of *PtCP1*, the specific spacer sequences (gRNA1, gRNA2, and gRNA3) were selected using a CCTop-CRISPR/Cas9 target online predictor (https://cctop.cos.uni-heidelberg.de (accessed on 1 June 2022)). Transgenic poplar plants were generated by *A. tumefaciens-*mediated transformation. The materials were selected using hygromycin B. Total RNA was extracted from the samples after transfection and then used for amplifying *PtCP1* full-length cDNA and sequencing.

### 2.11. Laser Confocal Microscopy Experiment

Full-length cDNA (without TGA) was cloned to create the PtCP1–GFP–pCAMBIA 1300 fusion construct. The acid organelle stain Lysotracker Red was used. Transgenic tobacco leaf epidermis cells were visualized using an LSM880 confocal laser scanning microscope (Zeiss, Oberkochen, Germany). GFP was excited with an argon laser at a wavelength of 488 nm, and emission was detected at 500 nm and 530 nm. Chloroplast autofluorescence was excited with an argon laser at a wavelength of 488 nm, and emission was detected at 650 nm and 750 nm.

### 2.12. Measurement of Photosynthetic Parameters

Maximal PSII quantum yield (F_v_/F_m_) values were measured using a DualPAM-100 measuring system (Walz, Effeltrich, Germany) in a glass cuvette under agitation after 20 min of dark adaptation. The Fo (minimum fluorescence yield) was measured under weakly modulated measuring light (10 µmol photons m^−2^ s^−1^), while the Fm (maximum fluorescence yield) was measured by applying a saturating pulse of white light (4000 mmol photons m^−2^ s^−1^ for 0.8 s). F_v_/F_m_ was calculated from the ratio (F_m_ − F_o_)/F_m_. The CO_2_ response (A-C_i_) curve and the net photosynthetic rate were measured using a portable photosynthesis system (Li-Cor 6800; Li-Cor Inc. Lincoln, USA). Net assimilation, g_sw_, and C_i_ measurements were made under the following conditions using the 12th leaves from top to bottom: light intensity of 1200 µmol m^−2^ s^−1^, leaf temperature of 30 °C, and 50% humidity. A-C_i_ curves were measured in the light intensity of 1200 µmol m^−2^ s^−1^, leaf temperature of 30 °C, response curves were initiated using the following sequence of reference CO_2_ concentrations:400, 300, 200, 120, 70, 30, 10, 400, 400, 500, 700, 900, 1200, and 1500 µmol m^−2^ s^−1^ [25].

### 2.13. Transcriptome Analysis

Total RNA was extracted from 2-month-old WT, EM, and d7 leaves. RNA integrity was assessed using the RNA Nano 6000 Assay Kit of the Bioanalyzer 2100 system (Agilent Technologies, Santa Clara, CA, USA). Reference genome sequences of *Populus trichocarpa* were retrieved from the Ensembl Genomes database, release 23 (plants), available at ftp://ftp.ensemblgenomes.org/pub/release-23/plants/fasta/populus_trichocarpa/dna/. The primary assembly FASTA file was used for read mapping. The clustering of the index-coded samples was performed on a cBot Cluster Generation System using TruSeq PE Cluster Kit v3-cBot-HS (Illumia) according to the manufacturer’s instructions. After cluster generation, the library preparations were sequenced on an Illumina Novaseq platform and 150 bp paired-end reads were generated. Differential expression analysis was performed using the DESeq2R package (1.20.0). Genes with an adjusted *p*-value ≤ 0.05 found by DESeq2 were assigned as differentially expressed. The functional enrichment analysis of DEGs was conducted using KEGG and GO databases. Sequencing of nine libraries (three biological replicates per genotype: WT, d7, and EM) yielded high-quality data, with Q20 > 97% and Q30 > 93% for all samples. Clean reads were aligned to the genome, achieving overall mapping rates > 70% and uniquely mapped rates > 69%. These metrics confirm the suitability of the data for further analysis.

## 3. Results

### 3.1. Characterization and Expression Pattern of AALP-like Papain PtCP1

**Figure 1:**
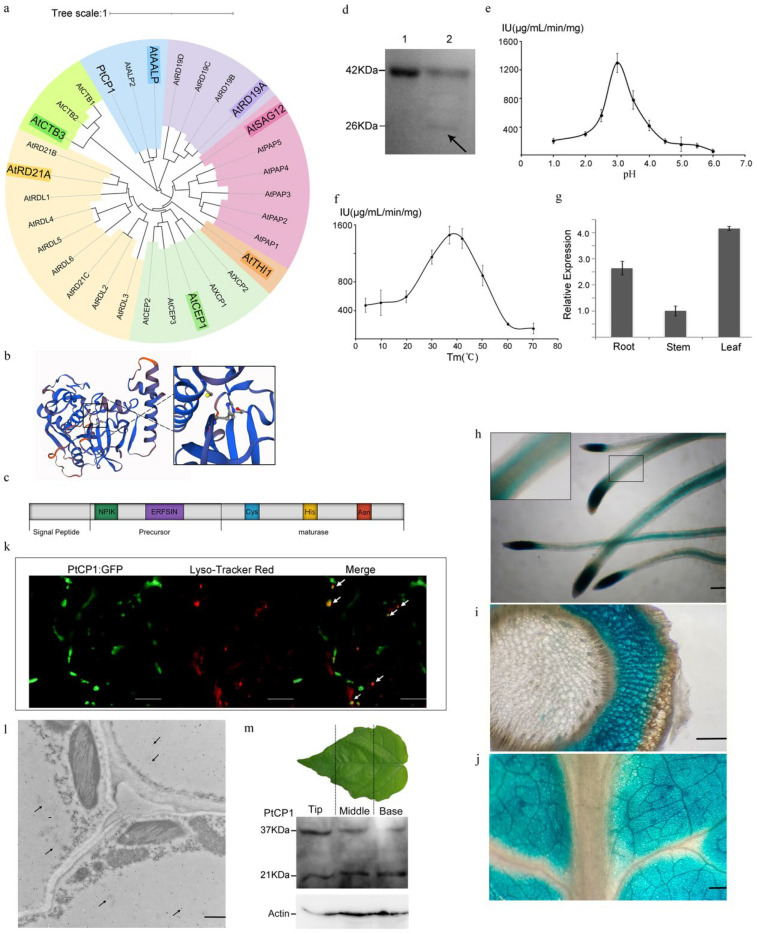
Characterization and expression pattern of PtCP1. (**a**) Phylogenetic tree analysis of *PtCP1* based on the neighbor-joining method with 1000 bootstrap replicates among 49 PLCP subfamily proteins. (**b**) Predicted three-dimensional structure of the PtCP1 protein. The black boxed region highlights the Cys-His-Asn catalytic triad of the protease. Swiss-model (https://swissmodel.expasy.org/) was used for the tertiary structure prediction and analysis. (**c**) Schematic diagram of PtCP1 gene structure. (**d**) Immunoblot analysis of PtCP1 zymogen activation. Lane 1: purified recombinant protein; Lane 2: zymogen and mature enzyme at pH 3.0. The black arrow indicates the mature enzyme. The recombinant protein was expressed in the *E. coli* BL21 (DE3) strain. Optimum pH (**e**) and optimum temperature (**f**) for PtCP1 mature enzyme. (**g**) Expression levels of *PtoCP1* across different organs of *P. tomentosa* via RT-qPCR. Organs (roots, stems, and leaves) were harvested from three individual 3-month-old wild-type poplar trees and pooled separately by tissue type. Error bars indicate ±SD derived from three biological replicates. (**h**–**j**) Histochemical assay for GUS activity in 1-month-old transgenetic tobacco roots (**h**), the cross section of stem (**i**), and leaves (**j**). Scale bar = 250 μm. (**k**) Subcellular localization of PtCP1 in 1-month-old transgenetic tobacco leaf epidermal cells. GFP fluorescence co-localizes with Lysotracker Red–labeled acidic organelles (white arrows). Scale bar = 25 μm. (**l**) Subcellular localization of PtCP1 revealed by immunogold labeling with a rabbit anti-PtCP1 antibody. Arrows indicated gold particle labeling. Scale bar = 500 nm. (**m**) Immunoblot detection of PtCP1 in 3-month-old wild-type poplar. Total proteins were extracted from the top, middle, and bottom regions of the 5th and 6th leaves counted from the apex.

The *P. trichocarpa* genomics database was searched, and a 1077 bp CDS (coding sequence) (Gen bank accession Number: XM_006381559.1) was cloned from *P. tomentosa Carr.* and named as *PtCP1*. According to sequence homology, PtCP1 was a papain-like protease (also called cathepsin H-like protease), which encoded 358 amino acids including a signal peptide of 22 amino acids. Furthermore, a phylogenetic tree showed that PtCP1 shared high homology with AALP-like subclasses and belonged to the 8th PLCP subfamily (AALP-like) [5,26] (Figure 1a).

ERFNIN (EX3RX3FX2NX3I/VX3N) exists in N-terminal propeptides of PtCP1, which is strongly conserved and seems to act to block the enzyme’s catalytic site, inhibiting activity and having proper folding and targeting [27]. The conserved catalytic triad, Cys-His-Asn, which is necessary for proteolytic activity, was also found in PtCP1 (Figure 1b). A vacuolar targeting sequence NPIK, which is only found in AALP-like proteases but not in any of the other PLCP subfamilies, was found at the N terminus of PtCP1, indicating that PtCP1 might function in the vacuole (Figure 1c).

We expressed the PtCP1 protein (without the signal peptide) in *E. coli* BL21 to further analyze the catalytic characteristics of PtCP1. The purified zymogen was 37 kDa without determinable activity, and it transformed into a 21 kDa mature protein by self-cleaving at pH 3.0 (Figure 1d) The kinetics of the mature enzyme were measured using casein as the substrate. The *K*_m_ was 32.18 ± 1.66 μg·mL^−1^, while *V*_m_ was 5105.80 ± 407.30 μg·mL^−1^·min^−1^. The optimum pH and temperature for the PtCP1 mature enzyme were pH 3.0 and 37 °C, respectively (Figure 1e,f). In addition, PtCP1 was inhibited significantly by the papain-specific inhibitor E-64. These results revealed that PtCP1 was an AALP-like papain with zymogen activation in an acidic environment.

Analysis was performed to explore the spatial localization of PtCP1, qRT-PCR (quantitative reverse transcription polymerase chain reaction), and the result showed that PtCP1 was expressed in leaves, stems, and roots, with the highest expression in leaves (Figure 1g). In addition, a 2270 bp promoter of *PtCP1* was cloned and inserted into the pBI121 vector for the pro*PtCP1*::GUS fusion constructs and then transfected into tobacco. GUS activity was detected in the mesophyll of leaves and phloem and cortex of roots and stems. (Figure 1h–j).

The subcellular localization of PtCP1 was investigated by fusing its full-length coding sequence upstream to the green fluorescent protein (GFP). GFP fluorescence was detected in the acid vacuole, which was confirmed by staining with Lysotracker Red (acid vacuole fluorescence marker) (Figure 1k). However, no fluorescence signal was observed in the CV (central vacuole). Considering that the fluorescence of vacuole-targeted GFP usually disappeared under light conditions caused by the degradation of GFP by vacuolar cysteine proteases [28], immunogold labeling was conducted using the rabbit anti-PtCP1 antibody. The result showed that PtCP1 was located in the vacuole of *P. tomentosa*, but not in the chloroplast (Figure 1l). These results suggested that the PtCP1 protein was localized to the CV and may mature in the acidic vacuole.

Contrary to earlier reports that cysteine protease induction is exclusive to leaf senescence, our immunoblotting analysis of PtCP1 revealed its early activation in developing leaves. We assessed the zymogen and mature enzyme in the base (tender), middle (maturing), and tip (more mature) leaf sections to establish a temporal activation profile. The results showed that the formation of mature enzyme was continuously accompanied throughout leaf development, with abundant proenzyme accumulated in the tip (more mature) leaves (Figure 1m), indicating that PtCP1 expression increased progressively during leaf senescence.

Taken together, our data indicated that PtCP1 was ubiquitously expressed and localized to the vacuole, where its acidic autoactivation enabled a functional role spanning from leaf development to senescence.

### 3.2. Early Maturation and Loss of PtCP1 Protease Activity Reveals Its Central Rrole in Regulating Growth

To further investigate the function of PtCP1 and inspired by its intriguing acid-dependent activation, we sought to generate both loss-of-function mutants and constitutively active mutants with ERFNIN domain deletion. Based on the principle of MMEJ repair (efficient CRISPR/Cas9-based plant genomic fragment deletions by microhomology-mediated end joining [29]), we designed a CRISPR-Cas9 construct with three sgRNAs targeting the flanking regions of the self-inhibitory ERFNIN domain. This design was intended to leverage two distinct cellular repair pathways simultaneously: the error-prone NHEJ (non-homologous end joining) pathway to generate small indels for loss-of-function mutants, and the MMEJ (microhomology-mediated end joining) pathway to create a precise in-frame deletion for the constitutively active mutant.

**Figure 2:**
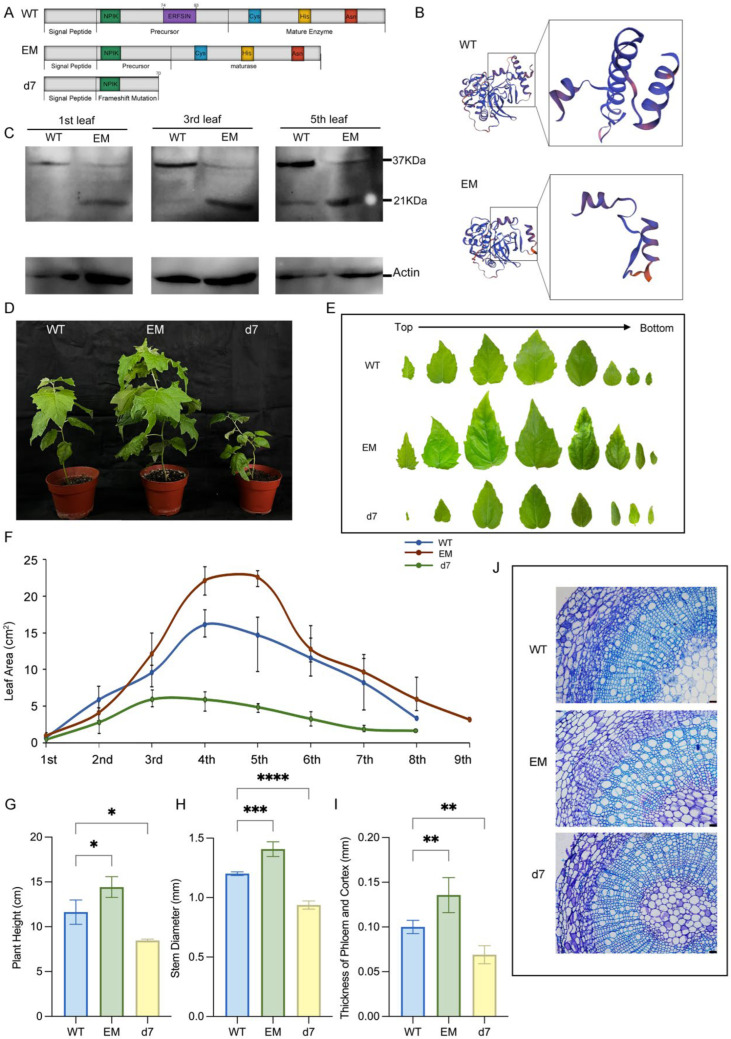
Phenotypic characterization of PtCP1 loss-of-function (d7) and early maturation (EM) transgenic poplar lines. *d7* was a dwarf with a thinner stem and smaller leaves. In contrast, the early-maturation transgenic plant exhibited the opposite phenotype. (**A**) Premature termination of PtCP1 in the *d7* line. (**B**) Predicted 3D structure of PtCP1 in EM and WT lines. Black box highlights the deleted ERFNIN domain. Swiss-model (https://swissmodel.expasy.org/) was used for the tertiary structure prediction and analyzation. (**C**) Immunoblotting of the zymogen and mature PtCP1 in the 1st, 3rd, and 5th leaves of 3-month-old *Populus*. The plant height (**D**,**G**), leaf area (**E**,**F**) of 2-month-old WT and transgenic lines. (**J**) Transverse sections through the second aboveground internode of WT and transgenic 2-month-old plants. Sections were stained with 0.01% (*w*/*v*) toluidine blue O. Scale bar = 100 μm. Stem diameter (**H**) and thickness of phloem and cortex (**I**) of 2-month-old plants. In (**F**–**J**), data were presented as mean ± SD from three biological replicates. Statistical significance was assessed using Student’s *t*-test (* *p* < 0.05; ** *p* < 0.01; *** *p* < 0.001; **** *p* < 0.0001).

First, we obtained a loss-of-function line, designated *d7*. As anticipated from the NHEJ pathway, this mutant contained small indels (a ‘T’ insertion at position 176 and a ‘CACCAATA’ deletion at positions 273–280) at the target sites. These indels caused a frameshift mutation, yielding a truncated, non-functional 100-aa polypeptide that lacked the catalytic triad (Figure 2A).

Second, conventional overexpression was unsuitable for probing the function of the mature enzyme directly, as the resulting protease would still be produced as a zymogen subject to the native acid activation checkpoint. Therefore, we successfully obtained the EM (‘early matured’) line. This line resulted from the hypothesized MMEJ pathway, which caused a precise 72 bp deletion encompassing the entire self-inhibitory ERFNIN domain. This deletion, triggered by the simultaneous DSBs, creates a protease that bypasses the native acid activation checkpoint (Figure 2A,B).

Immunoblotting was used to investigate the zymogen and mature enzyme in the 1st, 3rd, and 5th leaves of 3-month-old WT and EM lines, which represented the new, maturing, and matured leaf, respectively. In the WT, the proenzyme existed in new leaves, indicating PtCP1 was expressed in the early stages of leaf formation. Then, parts of the zymogen were gradually transformed into 21 kDa mature proteins with the development of leaves (Figure 2C). In contrast to the WT, the EM line accumulated the mature PtCP1 enzyme at earlier developmental stages and in greater abundance, confirming precocious activation of the protease.

Phenotypically, the constitutive activation of PtCP1 in the EM line promoted robust plant growth, whereas the *d7* mutant exhibited a severe dwarf phenotype (Figure 2D). Quantitative measurements showed that the EM line had significant increases in plant height (45% max), leaf area (40%), and stem diameter (16%) compared to WT (Figure 2E–H). Anatomical analysis attributed the thicker stems in EM primarily to a 37% expansion of the phloem and cortex (Figure 2I,J). Conversely, the *d7* mutant showed reductions in plant height (41%), stem diameter (22%), and leaf area, which correlated with a 31% thinning of the phloem and cortex (Figure 2E–J), underscoring the role of PtCP1-mediated proteolysis in sustaining tissue growth.

### 3.3. PtCP1-Mediated Rubisco Turnover Determines Photosynthetic Carbon Fixation

**Figure 3:**
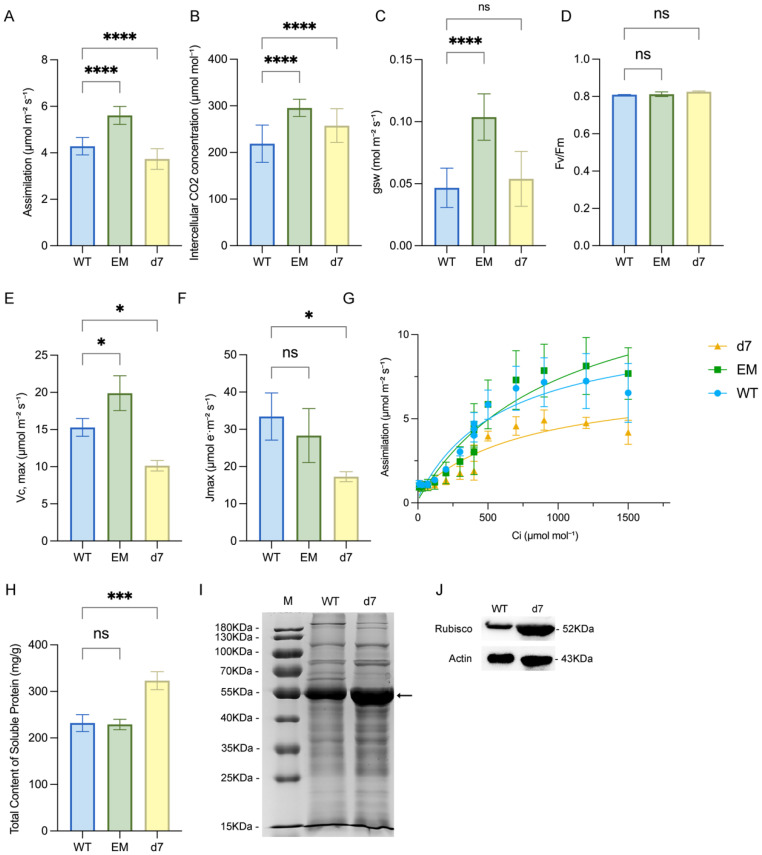
Physiological and biochemical analyses of photosynthetic performance and Rubisco accumulation in WT, EM, and *d7* plants Net photosynthetic rate ((**A**), P_n_), intercellular CO_2_ concentration ((**B**), C_i_), and stomatal conductance ((**C**), g_sw_) in 2-month-old plants. Measurements were made under the following conditions using the 12th leaves from top to bottom: light intensity of 1200 µmol m^−2^ s^−1^, leaf temperature of 30 °C, and 50% humidity. Data were presented as mean ± SD (n = 3). (**D**) Maximum photochemical efficiency of PSII (F_v_/F_m_). After being kept for at least 20 min, 2-month-old plants were measured using a DualPAM-100 system (Walz, Germany). (**E**,**F**) Maximum in vivo Rubisco carboxylation rate (V_c,max_) and maximum rate of RuBP regeneration (J_max_) at 30 °C estimated from response curves. Data were presented as mean ± SD (n = 3). Significance testing was conducted using Student’s *t*-test (* *p* < 0.05; *** *p* < 0.001; **** *p* < 0.0001; ns indicates no significant difference). (**G**) Response of light-saturated CO_2_ assimilation rates (**A**) to intercellular CO_2_ (A-response curve). Measurements were made under light intensity of 1200 μmol m^−2^ s^−1^, leaf temperature of 30 °C, CO_2_ were varied from 10 to 1500 µmol mol^−1^. Data are presented as mean ± SD (n = 3). (**H**,**I**) Total content of soluble proteins in mature leaves of 2-month-old plants. The arrow indicates the position of the Rubisco large subunit in line *d7*. (**J**) Immunoblot analysis of Rubisco content in mature leaves (5th–9th from top to bottom) of 2-month-old plants.

In order to understand the mechanism of PtCP1 improving tissue growth, we quantified key photosynthetic parameters. The EM line exhibited increases in the net photosynthetic rate (A, +30.87%), while the *d7* mutant showed a 12.87% decrease (Figure 3A). To determine whether the observed changes in net photosynthetic rate (A) in the EM and *d7* lines originated from alterations in the light or dark reactions of photosynthesis, we measured intercellular CO_2_ concentration (C_i_), stomatal conductance (g_sw_), and the maximum quantum efficiency of PSII (F_v_/F_m_). In the EM line, the increase in A was accompanied by a substantial rise in g_sw_ (+122.03%) and a concurrent increase in C_i_ (+35.07%), suggesting that enhanced stomatal opening improved CO_2_ availability, thereby synergistically promoting carbon assimilation (Figure 3B,C). By contrast, the *d7* mutant exhibited a 17.72% increase in C_i_ and no change in g_sw_, indicating that its photosynthetic limitation was not caused by stomatal constraints but rather by a decline in the efficiency of mesophyll-level dark reactions. Furthermore, the absence of significant changes in F_v_/F_m_ in both genotypes (Figure 3D) ruled out photoinhibition or damage to PSII, consolidating the conclusion that the photosynthetic defect in was *d7* localized to carbon fixation and Calvin cycle activity in the dark reactions.

Analysis of A-C_i_ curves showed a slightly enhanced maximum assimilation in EM but a strongly suppressed curve in *d7* compared to WT (Figure 3G). This was consistent with derived parameters: V_c,max_ increased by 30.08% in EM but decreased by 33.69% in *d7*, whereas J_max_ was unchanged in EM but plummeted by 48.39% in *d7* (Figure 3E,F). This demonstrated that the photosynthetic gain of EM was linked to superior CO_2_ fixation, whereas *d7*’s defect stems from concurrent failures in both carboxylation and regeneration phases of the Calvin cycle.

Given the established role of cysteine proteases in protein degradation and the observed decline in photosynthetic capacity in the *d7* mutant, we hypothesized that PtCP1 deficiency might disrupt the turnover of photosynthetic proteins, leading to their aberrant accumulation and consequently impairing photosynthetic function. To test this, we first assessed the total leaf soluble protein content, which revealed a significant 39% increase in the *d7* line (Figure 3H). Subsequent immunoblot analysis confirmed a specific accumulation of the large subunit of Rubisco (RbcL, 52 kDa) (Figure 3I,J). This finding indicated that the loss of PtCP1 function impaired the proteolytic turnover of proteins, including key photosynthetic components like Rubisco. The resulting accumulation of undegraded protein, occurring alongside a measured decline in photosynthetic capacity, underscored a critical role for PtCP1 in maintaining protein homeostasis during leaf development and senescence. We postulated that this disruption in Rubisco degradation and the consequent decline in carbon fixation efficiency would lead to a fundamental imbalance in carbohydrate metabolism. To test this hypothesis and uncover the associated transcriptional changes, we conducted genome-wide transcriptome profiling of the transgenic plants.

Transcriptome analysis revealed reprogramming of Carbon Metabolism in *d7* line.

**Figure 4:**
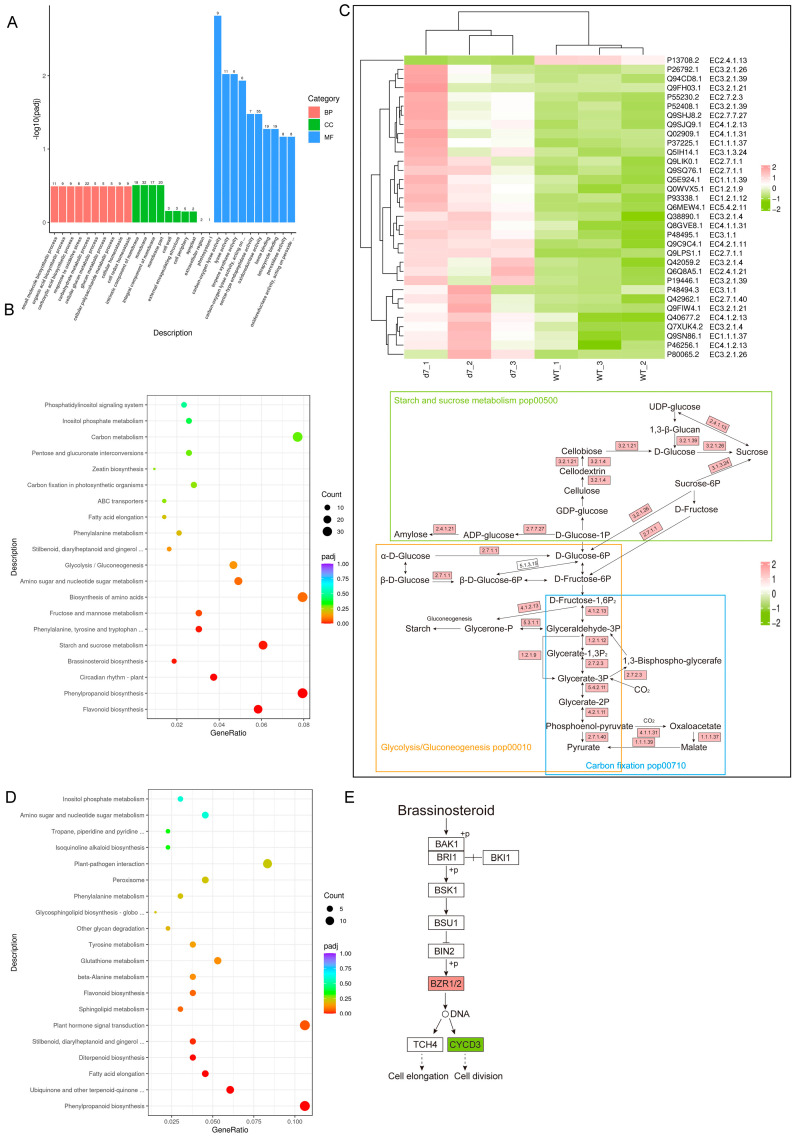
Transcriptome analysis of EM and *d7* line. Total RNA was extracted from 2-month-old WT, EM, and *d7* leaves (n = 3 biological replicates per genotype). Differentially expressed genes (DEGs) were identified using a cutoff of |fold change| ≥ 1 and *p*adj ≤ 0.05. (**A**,**B**) GO and KEGG enrichment analyses of DEGs between WT and *d7* line. (**C**) Heatmap displaying gene expression profiles (log2-fold change relative to WT) of metabolic genes (labeled with locus IDs and EC numbers) across *d7*-1, *d7*-2, *d7*-3, and WT lines. Color intensity (red: upregulation; green: downregulation) indicated expression magnitude. Integrated map of starch/sucrose metabolism (ko00500), glycolysis/gluconeogenesis (ko00010), and carbon fixation (ko00710) pathways, overlaid with the corresponding gene expression changes (labeled with EC numbers) in the *d7* line. Pathway modules were demarcated by colored boxes (green: starch and sucrose metabolism; orange: glycolysis; blue: carbon fixation). (**D**) KEGG enrichment analyses of DEGs between WT and EM. (**E**) The diagram illustrated the core signaling cascades of cytokinin (left) and brassinosteroid (BR, right) pathways in EM (red: upregulation; green: downregulation).

To further elucidate the roles of PtCP1 in photosynthetic efficiency, we conducted RNA-seq analysis to assess gene expression profiles in the EM, *d7,* and WT. Compared to the wild-type (WT), *d7* exhibited 719 differentially expressed genes (DEGs), with 284 upregulated and 435 downregulated. Gene Ontology (GO) analysis showed that these DEGs were significantly enriched in processes such as carbohydrate metabolic process, carboxylic/organic acid biosynthetic processes and carbon-oxygen lyase activity (Figure 4A). KEGG (Kyoto Encyclopedia of Genes and Genomes) pathway analysis further revealed predominant enrichment in carbon metabolism, amino acid biosynthesis, starch and sucrose metabolism, and carbon fixation in photosynthetic organisms (Figure 4B). Heatmaps and metabolic pathway diagrams illustrated that key enzymes in starch and sucrose metabolism, glycolysis/gluconeogenesis, and carbon fixation were largely upregulated in *d7* (Figure 4C). This transcriptional pattern suggested a state of metabolic dysregulation in *d7*. The upregulation of carbon fixation and utilization pathways, despite the observed reduction in photosynthetic efficiency (Figure 3), implied an intracellular compensation for inefficient carbon use or a disruption in carbon allocation. This failure to efficiently translate fixed carbon into growth aligns with the severe dwarf phenotype of *d7*. In other words, the developmental delay in the *d7* mutant arose from disruptions in both sugar and carbon metabolism.

In contrast, transcriptome analysis of the EM line identified 620 DEGs relative to WT, including 362 upregulated and 258 downregulated genes. GO analysis indicated enrichment in organic acid biosynthetic processes, response to stress, carboxylic acid metabolic processes, and transferase activity. KEGG enrichment highlighted pathways such as plant hormone signal transduction, phenylpropanoid biosynthesis, and response to plant-pathogen interaction (Figure 4D). Unlike *d7*, transcriptomic changes in the EM line were not enriched in central metabolism but in plant hormone signaling, particularly the brassinosteroid (BRs) pathways (ko04075) (Figure 4E). BRs have been reported to regulate initial chloroplast development, which is mediated by the transcription factor BRASSINAZOLE RESISTANT1 (BZR1) and its homologue BZR2/BES1; both form homodimers to control transcription, exerting significant effects on chloroplast development and photosynthesis [30,31]. Studies have shown that enhancing endogenous BR biosynthesis specifically in vegetative tissues enlarges the sugar pools in flag leaves and promotes grain filling and yield in rice [32]. Furthermore, PagBZR1 was reported to interact with GROWTH-REGULATING FACTOR 5 (PpnGRF5), and their interaction delayed chlorophyll degradation [31]. Overexpression of PpnGRF5 resulted in larger leaves and higher photosynthetic rates [33]. Our results revealed that the superior photosynthesis in the EM line was orchestrated by BR-led hormonal reprogramming, rather than metabolic dysfunction as seen in *d7*. This shift to a hormonally regulated strategy underpins its robust growth. A qRT-PCR validation of gene expression in the EM and d7 lines showed trends consistent with the RNA-seq data (Supplementary Figure S2).

Our comprehensive analysis demonstrated that PtCP1 protease activity played a pivotal role in regulating plant growth and development in *P. tomentosa*. Genetic evidence from the CRISPR-Cas9-generated mutants revealed that constitutive activation of PtCP1 in the EM line promotes robust growth, enhancing photosynthetic capacity, stem thickness, and overall biomass. Conversely, the loss-of-function *d7* mutant exhibited a severe dwarf phenotype accompanied by profound disruptions in carbon metabolism. The contrasting phenotypes of the EM and *d7* lines were correlated with distinct transcriptomic signatures. The EM transcriptome was characterized by a reprogramming of hormone networks, favoring pathways that drive cell elongation and differentiation, which underlies its accelerated growth. In contrast, the *d7* mutant was marked by widespread disruption in the expression of genes central to carbohydrate metabolism and carbon fixation. Crucially, the observed non-stomatal limitation to photosynthesis in *d7*, characterized by reduced carboxylation efficiency coupled with aberrant accumulation of Rubisco and soluble proteins, established PtCP1 as a critical regulator maintaining the balance between protein homeostasis and photosynthetic carbon utilization. These findings collectively positioned PtCP1 at the nexus of proteolysis, carbon metabolism, and plant growth control.

## 4. Discussion

Protein degradation and recycling are critical to plants. We identified a PLCP, PtCP1, which was highly expressed in the leaves of *P. tomentosa*. PtCP1 acted as a crucial executor for protein degradation during the vegetative growth and senescence stage.

Previous reports showed that premature protease was transported to protease vesicles, presumably via the endoplasmic reticulum; transported to the vacuoles, ricinosomes, or lysosomes; and transformed into mature protease by self-cleavage or protease-dependent maturation [34]. The transport and localization of protease in cells mainly depends on its localization signal sequence. Different subcellular organelles are also conducive to the maturation of different proteases. Because the NPIR motif is a vacuolar targeting signal [35], the N-terminal NPIK motif in PtCP1 indicated that it may function in the vacuole. The recombinant pro-PtCP1 underwent self-hydrolysis in vitro at pH 3.0, which also further supported the localization of PtCP1 in acidic vacuoles, not in ricinosomes or lysosomes, with pH 4–6.

*Arabidopsis* cysteine proteases, such as CEP1, βVPE, and γVPE, were located in vacuoles as proenzymes. During PCD (programmed cell death), the proenzyme was transformed into the mature enzyme and played an important role in the xylem and tapetum [23,36]. However, mesophyll cells and phloem did not undergo the obvious PCD process during vegetative growth. Thus, PtCP1, with specific expression in the mesophyll of leaves and phloem, functioned in acid vacuoles but not in CVs. Using immunocolloidal gold and GFP fluorescence localization, we successfully observed the localization of PtCP1 in the CV and the small acid vacuole.

These results showed that PtCP1 was expressed specifically in the mesophyll of leaves and phloem during plant growth, first localized in vacuoles as a proenzyme, and may then be transported to the acid vacuole, where it is presumably converted into the mature enzyme at pH 3.0 to participate in protein degradation.

The chloroplast is the main site of photosynthesis and is indispensable for plant growth and development. About >70% of total cellular nitrogen is located in mesophyll chloroplasts and is remobilized during leaf senescence [37]. The possible pathways of chloroplast breakdown are autophagy, SAVs (senescence-associated vacuoles), chloroplast vesiculation, and/or selective chloroplast destruction [38,39,40]. During leaf senescence, chloroplast proteins are first degraded by intraplastidial peptidehydrolases, and then chloroplasts or spherical bodies deriving from chloroplasts reach the vacuole for the final degradation by extraplastidial enzymes [41,42]. About 50 intraplastidial peptidehydrolases to date, which majorly include serine proteases, metalloproteases, and aspartyl proteases, such as Clp, FtsH, and DegP, to remove damaged, aggregated, dysfunctional, or mislocalized proteins [43,44]. The misexpression of a chloroplast aspartyl protease, NANA, leads to the dwarf phenotype and alters carbohydrate metabolism in *Arabidopsis* [15,45].

Most of the extraplastidial enzymes are located in SAVs, which are small, acidic vacuoles with high cysteine peptidase activity and are clearly different from the central vacuole. Rubisco, and other important stromal proteins, may be degraded mainly in SAVs, which are also named as “Rubisco vesicular bodies, RCBs.” RCBs accumulate in the cytosol of senescing leaves of wheat, *Arabidopsis*, and tobacco leaves [10,41,46,47]. The treatment of leaf disks with E-64 in vivo reduced the degradation of Rubisco and leaf proteins [48]. Other researchers believed that differences existed between RCBs and SAVs because SAVs were acidic compartments containing cysteine proteases [49]. However, in any case, both RCBs and SAVs contained a large number of Rubisco [18].

RVBs were observed in young leaves, indicating Rubisco protein turnover [10]. However, SAVs were undetectable in mature, nonsenescing leaves in *Arabidopsis* with the acidotropic vacuolar marker Lysotracker Red, but their number increased significantly in soybean, *Arabidopsis*, and tobacco leaves senescing in the dark [18]. In our study, much smaller, acidic vacuoles were detectable in nonsenescing leaves in *P. tomentosa* with the acidotropic vacuolar marker Lysotracker Red, suggesting that the degradation of chloroplasts in nonsenescing leaves of *P. tomentosa* might work in acidic vacuoles.

Rubisco catalyzes the initial CO_2_ fixation step in the Calvin–Benson–Bassham cycle, converting ribulose-1,5-bisphosphate into 3-phosphoglycerate (3-PGA)—a central intermediate that initiates carbohydrate synthesis in plants. Through sequential reduction and regeneration steps, 3-PGA is converted into triose phosphates, which are used either to regenerate RuBP in the chloroplast or to synthesize sucrose in the cytosol. Despite its essential role, Rubisco exhibits low catalytic efficiency, characterized by limited affinity for CO_2_ and a slow turnover rate. Considerable efforts have been made to improve photosynthetic performance by engineering Rubisco to enhance its carboxylation efficiency and CO_2_ fixation capacity. For instance, modulating the photorespiratory pathway increased tobacco biomass [50], and overexpressing the Rubisco small subunit in rice enhanced holoenzyme assembly and grain yield under sufficient nitrogen fertilization [51]. These results are consistent with a broader set of studies demonstrating the potential of Rubisco engineering to enhance carbon assimilation and plant growth [52,53,54,55,56,57,58,59,60]. In line with this, we found that impaired Rubisco turnover in the PtCP1 mutant (*d7*) led to RbcL accumulation, accompanied by significant decreases in photosynthetic and carboxylation efficiency. This indicated that PtCP1 regulated the dark reactions of photosynthesis by influencing Rubisco protein turnover, which subsequently affected sugar metabolism and plant growth. Conversely, the EM-PtCP1 line, with enhanced Rubisco degradation capacity, showed improved photosynthesis and carboxylation, supporting the role of PtCP1 in facilitating photosynthetic efficiency and vegetative growth. Proper regulation of PtCP1 activation was necessary for efficiency. It was a potential way to improve woody plant biomass.

In summary, this study tested the hypothesis that additional cysteine proteases, beyond those known in senescence, actively regulate protein turnover and carbon metabolism during vegetative growth in woody plants. Using Populus tomentosa, we identified and characterized PtCP1, an AALP-like cysteine protease specifically expressed in leaf mesophyll and phloem, and determined its subcellular localization, activation mechanism, and physiological impact. Our results show that: (i) PtCP1 is targeted to acidic vacuoles as a proenzyme and undergoes auto-activation at pH 3.0; (ii) it functions extraplastidially, mediating chloroplast protein degradation (notably Rubisco) in non-senescing leaves; and (iii) loss of PtCP1 impairs Rubisco turnover, reducing photosynthetic and carboxylation efficiency, whereas its early maturation enhances carbon assimilation and biomass accumulation. These findings validate our hypothesis and reveal a novel link between vacuolar cysteine protease activity and the dark reaction of photosynthesis. Importantly, Rubisco degradation in non-senescing leaves challenges the traditional view that substantial chloroplast breakdown is restricted to senescence; instead, *P. tomentosa* maintains active protein turnover during vegetative growth to optimize photosynthetic efficiency. Thus, PtCP1 represents a potential biotechnological target for improving woody plant productivity by modulating proteolysis-coupled carbon metabolism. Despite the limitation of using a single representative line per genotype, the consistent reciprocal phenotypes between loss-of-function and early-matured lines, together with multiple biological replicates and molecular validation, strongly support a role for PtCP1 in linking proteolysis to photosynthetic carbon fixation and biomass accumulation in poplar.

## Data Availability

The original contributions presented in this study are included in the article/Supplementary Materials. Further inquiries can be directed to the corresponding author.

## Supplementary Materials

The following supporting information can be downloaded at: https://www.mdpi.com/article/10.3390/plants15101530/s1, Table S1. Gene information and primers’ sequence used of qRT-PCR. Gene IDs were based on the *P. trichocarpa* v3.1 genome annotation, obtained from the Phytozome database. Figure S1. Quantification of PtCP1 zymogen (Z) and mature enzyme (M) protein levels in different leaf positions (corresponding to Figure 1m). Band intensities of the 37 kDa zymogen (Z) and the 21 kDa mature enzyme (M) were quantified using ImageJ software (ImageJ 1.53k). The signal of each band was normalized to the corresponding Actin loading control. Data are presented as mean ± SD from three independent biological replicates (n = 3). Figure S2. qRT-PCR assay showed that the expression level of *PtoPAL3/PtoGATL1/PtoPRX47/PtoIRX10/PtoERF017* in the leaves of 2-month-old EM line, and *Hexokinase/Alpha-amylasa/pfk/PGAM* in d7 line. Data are shown a mean ± s.d. from three biological replicates. Statistical significance was determined by Student’s *t*-test (*** *p* < 0.001). The corresponding gene ids were shown in Supplementary Table S1.

## References

[1] Dikic I. (2017). Proteasomal and Autophagic Degradation Systems. Annu. Rev. Biochem..

[2] Raffeiner M., Zhu S., Gonzalez-Fuente M., Ustun S. (2023). Interplay between autophagy and proteasome during protein turnover. Trends Plant Sci..

[3] Rawlings N.D., Barrett A.J., Thomas P.D., Huang X., Bateman A., Finn R.D. (2018). The MEROPS database of proteolytic enzymes, their substrates and inhibitors in 2017 and a comparison with peptidases in the PANTHER database. Nucleic Acids Res..

[4] Diaz-Mendoza M., Velasco-Arroyo B., Gonzalez-Melendi P., Martinez M., Diaz I. (2014). C1A cysteine protease-cystatin interactions in leaf senescence. J. Exp. Bot..

[5] van der Hoorn R.A. (2008). Plant proteases: From phenotypes to molecular mechanisms. Annu. Rev. Plant Biol..

[6] Huang J., van der Hoorn R.A.L. (2025). RD21-like proteases: Key effector hubs in plant-pathogen interactions. J. Exp. Bot..

[7] Cristofoletti P.T., Ribeiro A.F., Terra W.R. (2005). The cathepsin L-like proteinases from the midgut of Tenebrio molitor larvae: Sequence, properties, immunocytochemical localization and function. Insect Biochem. Mol. Biol..

[8] Karrer K.M., Peiffer S.L., DiTomas M.E. (1993). Two distinct gene subfamilies within the family of cysteine protease genes. Proc. Natl. Acad. Sci. USA.

[9] Alomrani S., Kunert K.J., Foyer C.H. (2021). Papain-like cysteine proteases are required for the regulation of photosynthetic gene expression and acclimation to high light stress. J. Exp. Bot..

[10] Prins A., van Heerden P.D., Olmos E., Kunert K.J., Foyer C.H. (2008). Cysteine proteinases regulate chloroplast protein content and composition in tobacco leaves: A model for dynamic interactions with ribulose-1,5-bisphosphate carboxylase/oxygenase (Rubisco) vesicular bodies. J. Exp. Bot..

[11] Prywes N., Phillips N.R., Tuck O.T., Valentin-Alvarado L.E., Savage D.F. (2023). Rubisco Function, Evolution, and Engineering. Annu. Rev. Biochem..

[12] Sharkey T.D. (2024). The end game(s) of photosynthetic carbon metabolism. Plant Physiol..

[13] Iniguez C., Capo-Bauca S., Niinemets U., Stoll H., Aguilo-Nicolau P., Galmes J. (2020). Evolutionary trends in RuBisCO kinetics and their co-evolution with CO_2_ concentrating mechanisms. Plant J..

[14] Busch F.A. (2020). Photorespiration in the context of Rubisco biochemistry, CO_2_ diffusion and metabolism. Plant J..

[15] Frank S., Hollmann J., Mulisch M., Matros A., Carrion C.C., Mock H.P., Hensel G., Krupinska K. (2019). Barley cysteine protease PAP14 plays a role in degradation of chloroplast proteins. J. Exp. Bot..

[16] Xiong Y., Contento A.L., Nguyen P.Q., Bassham D.C. (2007). Degradation of oxidized proteins by autophagy during oxidative stress in Arabidopsis. Plant Physiol..

[17] James M., Poret M., Masclaux-Daubresse C., Marmagne A., Coquet L., Jouenne T., Chan P., Trouverie J., Etienne P. (2018). SAG12, a Major Cysteine Protease Involved in Nitrogen Allocation during Senescence for Seed Production in *Arabidopsis thaliana*. Plant Cell Physiol..

[18] Otegui M.S., Noh Y.S., Martinez D.E., Vila Petroff M.G., Staehelin L.A., Amasino R.M., Guiamet J.J. (2005). Senescence-associated vacuoles with intense proteolytic activity develop in leaves of Arabidopsis and soybean. Plant J..

[19] Zhang X.M., Wang Y., Lv X.M., Li H., Sun P., Lu H., Li F.L. (2009). NtCP56, a new cysteine protease in *Nicotiana tabacum* L., involved in pollen grain development. J. Exp. Bot..

[20] Liu X., Mo L., Guo X., Zhang Q., Li H., Liu D., Lu H. (2021). How Cysteine Protease Gene PtCP5 Affects Seed Germination by Mobilizing Storage Proteins in Populus trichocarpa. Int. J. Mol. Sci..

[21] Yang R., Song J., Gu Z., Li C. (2011). Partial purification and characterisation of cysteine protease in wheat germ. J. Sci. Food Agric..

[22] Schmid M., Simpson D., Gietl C. (1999). Programmed cell death in castor bean endosperm is associated with the accumulation and release of a cysteine endopeptidase from ricinosomes. Proc. Natl. Acad. Sci. USA.

[23] Zhang D.D., Liu D., Lv X.M., Wang Y., Xun Z.L., Liu Z.X., Li F.L., Lu H. (2014). The cysteine protease CEP1, a key executor involved in tapetal programmed cell death, regulates pollen development in Arabidopsis. Plant Cell.

[24] Xie K., Minkenberg B., Yang Y. (2015). Boosting CRISPR/Cas9 multiplex editing capability with the endogenous tRNA-processing system. Proc. Natl. Acad. Sci. USA.

[25] Salesse-Smith C.E., Adar N., Kannan B., Nguyen T., Wei W., Guo M., Ge Z., Altpeter F., Clemente T.E., Long S.P. (2025). Adapting C(4) photosynthesis to atmospheric change and increasing productivity by elevating Rubisco content in sorghum and sugarcane. Proc. Natl. Acad. Sci. USA.

[26] Rawlings N.D., Barrett A.J., Bateman A. (2010). MEROPS: The peptidase database. Nucleic Acids Res..

[27] Beers E.P., Woffenden B.J., Zhao C. (2000). Plant proteolytic enzymes: Possible roles during programmed cell death. Plant Mol. Biol..

[28] Tamura K., Shimada T., Ono E., Tanaka Y., Nagatani A., Higashi S.I., Watanabe M., Nishimura M., Hara-Nishimura I. (2003). Why green fluorescent fusion proteins have not been observed in the vacuoles of higher plants. Plant J..

[29] Tan J., Zhao Y., Wang B., Hao Y., Wang Y., Li Y., Luo W., Zong W., Li G., Chen S. (2020). Efficient CRISPR/Cas9-based plant genomic fragment deletions by microhomology-mediated end joining. Plant Biotechnol. J..

[30] Cackett L., Luginbuehl L.H., Schreier T.B., Lopez-Juez E., Hibberd J.M. (2022). Chloroplast development in green plant tissues: The interplay between light, hormone, and transcriptional regulation. New Phytol..

[31] Chen H., Wu W., Du K., Ling A., Kang X. (2024). The interplay of growth-regulating factor 5 and BZR1 in coregulating chlorophyll degradation in poplar. Plant Cell Environ..

[32] Wu C.Y., Trieu A., Radhakrishnan P., Kwok S.F., Harris S., Zhang K., Wang J., Wan J., Zhai H., Takatsuto S. (2008). Brassinosteroids regulate grain filling in rice. Plant Cell.

[33] Wu W., Li J., Wang Q., Lv K., Du K., Zhang W., Li Q., Kang X., Wei H. (2021). Growth-regulating factor 5 (GRF5)-mediated gene regulatory network promotes leaf growth and expansion in poplar. New Phytol..

[34] Shimada T., Takagi J., Ichino T., Shirakawa M., Hara-Nishimura I. (2018). Plant Vacuoles. Annu. Rev. Plant Biol..

[35] Zhang X., Li H., Lu H., Hwang I., Gibbs D. (2021). The trafficking machinery of lytic and protein storage vacuoles: How much is shared and how much is distinct?. J. Exp. Bot..

[36] Han J., Li H., Yin B., Zhang Y., Liu Y., Cheng Z., Liu D., Lu H. (2019). The papain-like cysteine protease CEP1 is involved in programmed cell death and secondary wall thickening during xylem development in Arabidopsis. J. Exp. Bot..

[37] Onoda Y., Wright I.J., Evans J.R., Hikosaka K., Kitajima K., Niinemets U., Poorter H., Tosens T., Westoby M. (2017). Physiological and structural tradeoffs underlying the leaf economics spectrum. New Phytol..

[38] Ling Q., Broad W., Trösch R., Töpel M., Demiral Sert T., Lymperopoulos P., Baldwin A., Jarvis R.P. (2019). Ubiquitin-dependent chloroplast-associated protein degradation in plants. Science.

[39] Ling Q., Sadali N.M., Soufi Z., Zhou Y., Huang B., Zeng Y., Rodriguez-Concepcion M., Jarvis R.P. (2021). The chloroplast-associated protein degradation pathway controls chromoplast development and fruit ripening in tomato. Nat. Plants.

[40] McLoughlin F., Marshall R.S., Ding X., Chatt E.C., Kirkpatrick L.D., Augustine R.C., Li F., Otegui M.S., Vierstra R.D. (2020). Autophagy Plays Prominent Roles in Amino Acid, Nucleotide, and Carbohydrate Metabolism during Fixed-Carbon Starvation in Maize. Plant Cell.

[41] Chiba A., Ishida H., Nishizawa N.K., Makino A., Mae T. (2003). Exclusion of ribulose-1,5-bisphosphate carboxylase/oxygenase from chloroplasts by specific bodies in naturally senescing leaves of wheat. Plant Cell Physiol..

[42] Dominguez F., Cejudo F.J. (2021). Chloroplast dismantling in leaf senescence. J. Exp. Bot..

[43] Nishimura K., Kato Y., Sakamoto W. (2017). Essentials of Proteolytic Machineries in Chloroplasts. Mol. Plant.

[44] Kato Y., Murakami S., Yamamoto Y., Chatani H., Kondo Y., Nakano T., Yokota A., Sato F. (2004). The DNA-binding protease, CND41, and the degradation of ribulose-1,5-bisphosphate carboxylase/oxygenase in senescent leaves of tobacco. Planta.

[45] Paparelli E., Gonzali S., Parlanti S., Novi G., Giorgi F.M., Licausi F., Kosmacz M., Feil R., Lunn J.E., Brust H. (2012). Misexpression of a chloroplast aspartyl protease leads to severe growth defects and alters carbohydrate metabolism in Arabidopsis. Plant Physiol..

[46] Carrion C.A., Costa M.L., Martinez D.E., Mohr C., Humbeck K., Guiamet J.J. (2013). In vivo inhibition of cysteine proteases provides evidence for the involvement of ‘senescence-associated vacuoles’ in chloroplast protein degradation during dark-induced senescence of tobacco leaves. J. Exp. Bot..

[47] Ishida H., Yoshimoto K. (2008). Chloroplasts are partially mobilized to the vacuole by autophagy. Autophagy.

[48] Gomez F.M., Carrion C.A., Costa M.L., Desel C., Kieselbach T., Funk C., Krupinska K., Guiamet J. (2019). Extra-plastidial degradation of chlorophyll and photosystem I in tobacco leaves involving ‘senescence-associated vacuoles’. Plant J..

[49] Khanna-Chopra R. (2012). Leaf senescence and abiotic stresses share reactive oxygen species-mediated chloroplast degradation. Protoplasma.

[50] South P.F., Cavanagh A.P., Liu H.W., Ort D.R. (2019). Synthetic glycolate metabolism pathways stimulate crop growth and productivity in the field. Science.

[51] Yoon D.K., Ishiyama K., Suganami M., Tazoe Y., Watanabe M., Imaruoka S., Ogura M., Ishida H., Suzuki Y., Obara M. (2020). Transgenic rice overproducing Rubisco exhibits increased yields with improved nitrogen-use efficiency in an experimental paddy field. Nat. Food.

[52] Zhou Y., Whitney S. (2019). Directed Evolution of an Improved Rubisco; In Vitro Analyses to Decipher Fact from Fiction. Int. J. Mol. Sci..

[53] Whitney S.M., Von Caemmerer S., Hudson G.S., Andrews T.J. (1999). Directed mutation of the Rubisco large subunit of tobacco influences photorespiration and growth. Plant Physiol..

[54] Lin M.T., Occhialini A., Andralojc P.J., Parry M.A., Hanson M.R. (2014). A faster Rubisco with potential to increase photosynthesis in crops. Nature.

[55] Whitney S.M., Andrews T.J. (2001). Plastome-encoded bacterial ribulose-1,5-bisphosphate carboxylase/oxygenase (RubisCO) supports photosynthesis and growth in tobacco. Proc. Natl. Acad. Sci. USA.

[56] Occhialini A., Lin M.T., Andralojc P.J., Hanson M.R., Parry M.A. (2016). Transgenic tobacco plants with improved cyanobacterial Rubisco expression but no extra assembly factors grow at near wild-type rates if provided with elevated CO_2_. Plant J..

[57] Chen T., Riaz S., Davey P., Zhao Z., Sun Y., Dykes G.F., Zhou F., Hartwell J., Lawson T., Nixon P.J. (2023). Producing fast and active Rubisco in tobacco to enhance photosynthesis. Plant Cell.

[58] Zhao L., Cai Z., Li Y., Zhang Y. (2024). Engineering Rubisco to enhance CO(2) utilization. Synth. Syst. Biotechnol..

[59] Salesse-Smith C.E., Sharwood R.E., Busch F.A., Kromdijk J., Bardal V., Stern D.B. (2018). Overexpression of Rubisco subunits with RAF1 increases Rubisco content in maize. Nat. Plants.

[60] Salesse-Smith C.E., Sharwood R.E., Busch F.A., Stern D.B. (2020). Increased Rubisco content in maize mitigates chilling stress and speeds recovery. Plant Biotechnol. J..

