# Supplementary material for: PtCP1 Is an Extraplastidial Cysteine Protease Involved in Leaf Protein Degradation of Populus tomentosa Carr
Source: Plants (Basel). 2026 May 16;15(10):1530. doi: 10.3390/plants15101530 (PMC13211012; doi:10.3390/plants15101530)
Supplement: Supplementary file 1 [file plants-15-01530-s001.zip › plants-4278549-supplementary.pdf]

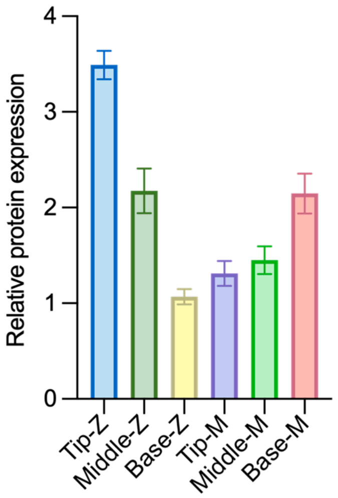

Supplementary Figure S1. Quantification of PtCP1 zymogen (Z) and mature enzyme (M) protein levels in different leaf positions (corresponding to Fig. 1M). Band intensities of the 37 kDa zymogen (Z) and the 21 kDa mature enzyme (M) were quantified using ImageJ software. The signal of each band was normalized to the corresponding Actin loading control. Data are presented as mean ± SD from three independent biological replicates (n = 3).

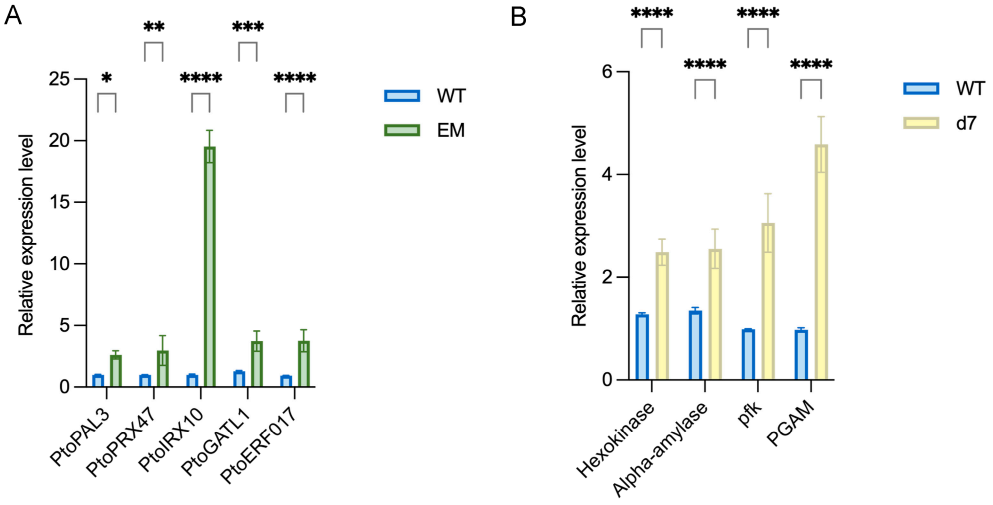

Supplementary Figure S2. qRT-PCR assay showed that the expression level of *PtoPAL3*/ *PtoGATL1*/ *PtoPRX47*/ *PtoIRX10*/ *PtoERF017* in the leaves of 2-month-old EM line, and *Hexokinase*/ *Alpha-amylasa*/ *pfk*/ *PGAM* in d7 line.Data are shown a mean ±s.d. from three biological replicates. Statistical significance was determined by Student's t-test (* $p < 0.05$; ** $p < 0.01$; *** $p < 0.001$; **** $p < 0.0001$). The corresponding gene ids were shown in Supplementary table S1.
